# Building trust: Leadership reflections on community empowerment and engagement in a large urban initiative

**DOI:** 10.1186/s12889-023-15860-z

**Published:** 2023-06-28

**Authors:** Amy E. Lansing, Natalie J. Romero, Elizabeth Siantz, Vivianne Silva, Kimberly Center, Danielle Casteel, Todd Gilmer

**Affiliations:** 1 Department of Psychiatry, University of California, San Diego, La Jolla, CA USA; 2 Department of Sociology, San Diego State University, San Diego, CA USA; 3 Herbert Wertheim School of Public Health and Human Longevity Science, University of California, San Diego, La Jolla, CA USA; 4 College of Social Work, University of Utah, Salt Lake, USA; 5 Department of Education and Information Studies, University of California, Los Angeles, Los Angeles, USA

**Keywords:** Trust-building, Trustworthiness, Community Capacity Building, Trauma Informed Practices, Equity, Community Empowerment, Community Engagement, Health Disparities, Adverse Childhood Experiences

## Abstract

**Background:**

Trust is essential for healthy, reciprocal relationships; creating safe environments; engaging in transparent interactions; successfully negotiating power differentials; supporting equity and putting trauma informed approaches into practice. Less is known, however, about the ways that trust-building may be at the forefront of consideration during community capacity building efforts, what trust-building elements are perceived as essential for optimally engaging communities, and what practices might support these efforts.

**Methods:**

The present study examines an evolving understanding of trust-building over the course of 3 years, from qualitative data derived during interviews with nine agency leads from a large and diverse urban community, who are spearheading community-based partnerships to create more trauma-informed communities and foster resiliency.

**Results:**

Data reflected fourteen trust-building elements, captured by three themes: 1) Building relationships and engagement (e.g., behavioral practices such as *meeting people* “*where they are at*” and *creating safe spaces*), 2) Embodying core values of trustworthiness (e.g., traits such as *being transparent* and *embodying benevolence*), and 3) Sharing decision-making, championing autonomy, and addressing barriers to trust (e.g., collaborative practices such as *creating a shared vision and goals* and *addressing systemic inequities*). These trust-building elements are presented in the Community Circle of Trust-Building, which provides an accessible, visual format that can facilitate capacity building efforts within organizations and with the broader community; guide the selection of training opportunities that support healthy interpersonal relationships; and aid in the identification of relevant, supporting frameworks (e.g., health equity, trauma-informed practices, inclusive leadership models).

**Conclusions:**

Community engagement and trust are essential for overall health and well-being, increasing equitable access to resources, and supporting an effective and connected citizenry. These data shed light on opportunities for trust-building and thoughtful engagement among agencies working directly with community members in large urban areas.

**Supplementary Information:**

The online version contains supplementary material available at 10.1186/s12889-023-15860-z.

## Background

Trust is essential for cultivating and sustaining healthy interpersonal relationships, facilitating civic engagement, fostering equitable access to resources, reducing health disparities, and promoting cooperation between local governing bodies and community members – all elements that are needed for effective societal functioning. Trust also facilitates relationship-building within large organizations, including service-based agencies such as developmental, mental health, medical and emergency services, that ideally represent the values, character, and goals of the individuals they serve [1, 2]. Public trust in governing bodies (also known as institutional trust) and interpersonal trust for representatives of governing agencies have been the subject of large-scale, multi-national surveys [3–6]. Historically, cross-country heterogeneity has been observed with *trust in others* ranging from 60%-86% in Scandinavian countries (e.g., Norway, Sweden, Denmark) to under 10% among some South American countries (e.g., Peru, Brazil) [3, 5]. Recent shifts towards political and economic austerity measures and authoritarianism around the world, coupled with a lengthy pandemic, eroded both institutional and interpersonal trust and increased partisan divides in many countries [7–10].

This pattern is evident in the United States (US) where few adults trust the government to do what is right ‘just about always’ (2%) or ‘most of the time’ (18%), 67% believe the government is not transparent (either ‘not much’ or ‘not at all’), and distrust is perceived as interfering with the country’s capacity to address contemporary problems [11–13]. Distrust in national and local governments results in service barriers (e.g., reduces help-seeking), is linked to poor mental and medical health outcomes, is associated with historical traumas and institutional betrayals and may be re-traumatizing [14–16]. Despite waning trust in the US [17], a recent international survey places the US as the sixth highest ranking country out of 28 countries for indicating trust in representatives of queried professions, such as: politicians in general, government representatives specifically, doctors, and scientists [18]. Taken together, these findings highlight the complexity of conceptualizing trust within and across countries.

Understanding trust at local levels is no less complex. The extant literature on trust spans disciplines (e.g., political, cognitive, and computer sciences, psychology, sociology, economics, risk management), industries (e.g., human resource management, marketing, business strategies, artificial intelligence), and initiatives (e.g., academic-community partnerships, public health endeavors). While there is no single intra- or inter-disciplinary definition of trust [19, 20], we approach trust in a similar fashion to Liu, Milojev, Gil de Zuniga and Zhang [2], conceptualizing trust within both a universal (having a broad, general context), and culture-specific, framework for understanding social relationships and interdependencies that include interpersonal, organizational and institutional levels. Trust is an incremental, ongoing process that involves choices based on the understanding that there are risks involved in making oneself vulnerable to, and/or dependent on others, and these vulnerabilities are not equally distributed among members of any given society [2]. Trust includes attitudes, beliefs, and behavioral intentions (“*willingness to be vulnerable*”) [21], not necessarily actual behaviors. Trust-building, however, involves behaviors and/or value-based characteristics (e.g., trustworthiness traits, such as transparency and reliability) that an individual or institution actively embodies and that promotes trust from others.

Further, being trustworthy is a core component of trauma-informed principles, which are designed to support the strengths of individuals while acknowledging the diversity of their life experiences and the multifaceted impact of adversity [22–25]. Implementation of trauma-informed principles, which includes providing information to a wide-range of stakeholders (e.g., parents, providers, teachers, community leaders, and agency staff) about the impact of trauma and embracing trauma-aware practices to improve well-being and service delivery, is increasingly common in both public agencies (e.g., behavioral health, juvenile justice, child welfare, public schools) and communities facing acute traumas and/or chronic, cumulative adversities such as poverty, mass incarceration and minority stress [26–29]. There is growing awareness of the magnitude of community trauma among mental health providers and institutional administrators, along with the recognition that traditional mental health services alone are unable to fully address the impact of trauma [30]. Recent work related to academic-community partnerships, public health initiatives with vulnerable communities, and community capacity building efforts suggest that trust is the essential first step for effective community engagement—particularly when power differentials create or amplify barriers to accessing needed services [31, 32].

Trust is also central to efforts to advance health equity through community engagement. In the US, large-scale efforts to improve health equity have amplified in the wake of the COVID-19 pandemic. The Equitable Long Term Recovery and Resilience federal initiative was developed to improve health equity through strategic networks and an emphasis on collaborative interagency (federal, regional/local, civilian) and community efforts across identified “vital conditions” necessary for health, population-level holistic well-being, and resiliency (e.g., basic health and safety needs, belonging/civic muscle, humane housing, reliable transportation) [33]. The initiative places trust at the center of engagement and genuine collaborative partnerships, and highlights some important elements of trust (e.g., transparency, demonstrating trust is warranted and will not be abused) but does not provide a more explicit roadmap for authentically building trust. The rich framework arising from this initiative does, however, provide guidelines to address social determinants of health and systemic inequities from a strengths-based perspective, support collective efficacy and bolster equitable recovery efforts while meaningfully engaging and empowering community members.

Similarly, the National Academy of Medicine's Leadership Consortium recently unveiled their integrated conceptual model for advancing health equity through community engagement and systemic changes to healthcare, which includes collaborative aims such as co-equality and shared governance [34]. Trust, both across partnerships and with the community, are considered necessary ingredients for partnership development, successful engagement and greater health equity in this framework, but the “how to” in terms of explicit guidelines for practices that support trust and trust-building, and the traits that may facilitate trust are not detailed. Finally, trust is a cornerstone of Community-Based Participatory Research initiatives such as Engage for Equity, whose goals are to strengthen partnerships and community collaboration through collective reflection and the co-creation of actionable knowledge (i.e., knowledge democracy), while developing tools and resources that support more equitable health-related decision making and creating an evidence base for measuring Community-Based Participatory Research outcomes [35–41].

While the importance of trust is clearly recognized across many sectors and large-scale initiatives, there are few explicit trust-building practice guidelines that inform public health efforts working with urban communities to co-identify needs as they emerge, reduce health disparities, increase health equity and improve resource access. Similarly, little is known about whether trust-building is at the forefront of consideration as agencies engage community members, implement trauma-informed practices on a wider scale, and work to improve access to resources that better reflect community members’ own perceptions regarding their unmet needs. The present study provides insight into leadership perspectives on community engagement and trust-building, as they emerged organically, during a large-scale, urban community capacity building initiative. We organized these findings to retain knowledge gained during the initiative, facilitate ongoing reflection and training in community-based initiatives, and foster conversations with community members.

We examined facets of trust-building derived from longitudinally collected semi-structured interviews with non-profit agency leads that spearheaded community-based partnerships designed to build community capacity aimed at reducing the negative impacts of trauma and fostering resiliency. This initiative occurred in Los Angeles County, the largest county in the United States, and included three waves of interviews planned to coincide with the initiative’s implementation, midpoint and final year. Contextually, the baseline, midpoint and final interviews occurred before the COVID-19 pandemic, during the height of the pandemic, and post-vaccination availability when restrictions were being lifted, respectively. Our objectives in this paper were to describe: 1) how trust organically emerged during the initial qualitative interviews, 2) how we approached a deeper understanding of the role of trust-building in community capacity building efforts during follow-up interviews spanning the initiative, and 3) how these findings may point to resources and training opportunities that guide future collaborative community embedded projects and research supporting sustainable public health equity efforts.

## Methods

### Project overview and setting characteristics

Innovations 2 was a community capacity building project designed by the Los Angeles County Department of Mental Health (DMH) to enhance community resiliency and promote community health from a trauma-informed perspective consistent with the core principles established by the Center for Disease Control and the Substance Abuse and Mental Health Services Administration: Safety; Trustworthiness and Transparency; Peer Support; Collaboration and Mutuality; Empowerment, Voice and Choice; and Cultural Competency (e.g., awareness of, and sensitivity to cultural, historical and gender considerations) [42]. Central goals included support for developing community leadership and fostering inter-agency and community collaborations in order to empower the community, expand the breadth of resources available to address community needs, and reach more community members. The project was not designed to offset mental health services, but rather to reach out and engage a broader cross-section of community members who have historically been disenfranchised and/or disinterested in directly engaging with mental health service agencies.

Nine partnerships, reflecting specific geographic regions representing the entirety of Los Angeles County, were funded to implement one or more of seven trauma-informed community capacity building strategies. Los Angeles County is the most populous county in the US, with a population larger than 40 states in the US and 9.86 million inhabitants [43]. It is also one of the geographically largest counties with over 4,000 square miles that include 88 incorporated cities and unincorporated areas. The population is diverse (49.1% Latinx, 25.3% White non-Latinx, 15.0% Asian, 7.9% Black, 2.3% Multiracial, 0.2% Native Hawaiian and Other Pacific Islander, 0.2% American Indian/Alaska Native), with over 56% of households speaking a language other than English in their homes, and 14.2% of the population living in poverty [44–47]. Capacity building strategies were unique to each community, intended for different demographic groups (e.g., transition age youth, parents of young children) with specific, supportive approaches to build skills, increase social connectedness, promote resource access and reduce the impact of adversity. All strategies included outreach and engagement efforts while providing links to resources and services for their community members, including attending to basic needs such as housing and food during the pandemic. Table 1 shows the regional representation of partnerships within Los Angeles County, the strategies employed, the target populations, numbers of partnering organizations, and numbers of trauma-informed trainings provided to partnership members, in support of their community capacity building efforts.

**Table 1:** Lead agency characteristics: strategy type, populations served, geographic regions, partners and training opportunities

**General service areas:**		Downtown LA (Pomona, Eagle Rock South Gate)	East LA (Boyle Heights)	Long Beach	Long Beach	Northeast LA (Pasadena)	Northern LA (Antelope Valley)	South LA	Venice/West LA (Marina del Rey, Mar Vista)	West LA (Santa Monica Culver City, Inglewood)	Number of partnerships using each strategy
***Strategy***	***Target population***										
Building Trauma Resilient Families	Caregivers with young children (0–5)	X	X		X			X		X	5
Trauma-informed Schools	Educators, school staff and families with school aged children: Head Start through High School	X	X					X		X	4
TAY Peer Support Network	16–25-year-old TAY		X	X		X	X		X		5
Coordinated Community Employment	Community members (TAY, adult) experiencing adversity, businesses supporting community members employment and finances			X							1
Community Re-entry & Reintegration	TAY / Adults with previous justice system involvement						X				1
Geriatric Empowerment	Intergenerational families, Older adults experiencing homelessness					X					1
Culturally Competent Support	Multigenerational families with systemic trauma exposure		X		X						2
Total # of Strategies	–	2	4	2	2	2	2	2	1	2	–
Total # of Partners*	–	8	6	20	31	29	7	21	10	11	–
Total # TIS Trainings	–	14.9	13.4	14.2	13.4	14.7	14.2	13.9	13.3	14.2	–

Several partnerships overlap regions but serve distinct populations or address different strategies: Alma and Para Los Ninos overlap in Downtown/East LA. MHMLA and TCC overlap in Long Beach and WIN and SPY overlap in Venice/West LA. *There are seven partnering agencies who are included in two different partnerships

*TAY* Transition Age Youth

### Ethics approval and consent to participate

All methods were carried out in accordance with relevant guidelines and regulations and were reviewed and approved by the University of California, San Diego Institutional Review Board (IRB #201892X). The data presented in this paper were previously collected as part of an evaluation of a program aiming to support partnerships in building community capacity to access resources and offset the impact of community trauma. The UCSD IRB determined that (a) the secondary use of the data for research purposes presented no more than minimal risk to human subjects; (b) the study qualified for review through the expedited procedure; and (c) it qualified for a waiver of informed consent.

### Study sample

Qualitative interviews were conducted with agency leads from nine community partnerships. Most partnerships were represented by a single person unless a partnership had separate leads representing different strategies or distributed duties. In cases with more than one agency lead, interviews were conducted separately. Agency leads were key informants knowledgeable about their agency, partnership, and strategies and were instrumental in decision-making and setting the tone for their partnership collaborations. Interviews were conducted with agency leads because they were familiar with the grant application goals; had a breadth and depth of knowledge about how their partnerships and strategies evolved over time; understood administrative and communication aspects of the project; knew how partnerships interacted with their unique communities; were instrumental in what types of training were provided across staff roles; and were key to the vision and mission of their partnerships.

Ten baseline interviews were conducted (80.0% female; one agency with two agency leads), with the following race/ethnicity composition: 30% Black; 30% Latinx; and 10% each Biracial/multi-ethnic, non-Latinx White and Greek. Eleven midpoint interviews were conducted (63.6% female, two agencies with two agency leads) with 36.4% Black; 18.2% each for Latinx and non-Latinx White; and 9.1% each Asian, Greek or Biracial agency leads. Ten final interviews were conducted (80.0% female; one agency with two agency leads) with 60.0% Latinx; 20.0% Black; 10.0% Asian, and 10% non-Latinx White agency leads.

### Development of interviews and data collection

Interview questions were crafted to address implementation considerations, emergent themes, changes, obstacles, impact, and sustainability over the course of the initiative. The three waves of interviews were planned to coincide with (a) the end of the first year of initiative implementation (baseline interview) to address the adaptations needed during the early phases of this learning initiative as grant application goals met the reality of community needs; (b) the end of the second year (midpoint interview) to explore themes that emerged during the baseline interview; assess pivots needed to adapt to and/or address COVID-19; and address impact and sustainability considerations; and (c) the initiative’s wrap-up (final interview) to understand how the initiatives approaches (e.g., learning-based project culture, trauma-informed practices, resiliency models) and large-scale challenges (e.g., pandemic, civil unrest) impacted how agency leaders envisioned sustainable community partnerships. Contextually, the baseline, midpoint and final interviews occurred before the COVID-19 pandemic, during the height of the pandemic, and post-vaccination availability when restrictions were being lifted, respectively.

One evaluation team member (a clinical psychologist) developed an initial set of questions for each of the three annual agency lead interviews. These initial question sets were then refined collaboratively by the evaluation team which represented a broad range of perspectives including staff and faculty trained in clinical psychology, health policy, social work, program evaluation, and public health. The final sets of questions included relevant prompts and follow-up questions to assist in consistent acquisition of information across interviewers. The annual interviews were conducted by MA and PhD trained members of the evaluation team who were familiar with the overall project goals and were experienced interviewers. In each year, interviewers debriefed after their first interviews, to refine questions and align follow-up questions as needed.

The baseline interview was conducted in-person (August and September of 2019) and constructed to explore how leadership perspectives on the initiative had evolved since submitting their grant applications. The focus of the baseline interviews was twofold: 1) to understand adaptations that agency leads made as they implemented their strategies following a delay in funding that required them to rethink the needs of their communities and which partners would best serve the strategies and communities associated with the initiative, and 2) to explore the nuts and bolts of such a complex undertaking (e.g., understanding adversity exposure in their communities, community engagement, managing communication). We specifically queried: 1) how they created a common language among partnership members and the community through shared definitions relevant to the project, 2) the trauma-types experienced by community members and what supports were most needed in their region; 3) partnership and strategy development and adaptation; 4) communication; and 5) learning and reflections about project implementation. Example questions included: *What does ‘community capacity building’ mean to you? When you think about “trauma” or “ACEs [Adverse Childhood Experiences]” in your community, what types of experiences come to mind? What were the most important factors that went in to selecting your partners? How do you typically communicate with your community? What does your partnership perceive as your community’s biggest challenge to overall well-being and health?* Neither trust nor trust-building were specifically queried.

The midpoint interview was conducted via Zoom (November and December of 2020) and addressed the project pivots that were occurring due to COVID-19 and shutdown orders, as well as changes in economic and resource availability, increased food insecurity/residential instability and discrimination-related civil unrest. The midpoint interview reflected on: 1) key themes that emerged organically during the baseline interview (relationship-building, *“meeting people in the community where they are at*”), 2) trauma informed approaches to community capacity building (e.g., principles of transparency, safety, trust); 3) establishing collaborative goals with community members and meeting their needs in light of COVID-19 and civil unrest; and 4) the project’s impact and sustainability (e.g., involving community members in decision-making, supporting existing community leaders, hiring and empowering community members with lived experience to spearhead engagement). Example questions included: *What are the biggest barriers to safety and trust in your community now? Which Innovation 2 project goals are most aligned with your community’s needs in response to COVID-19 and civil unrest? In what way has the Innovations 2 outreach changed how your community talks about trauma, adversity (e.g., stressful experiences) and their most pressing needs? How do you involve community members in conversations about sustaining trauma-informed community capacity building after Innovations 2?*

The Final Interview was conducted via Zoom (November 2021) and asked agency leads to reflect on Innovations 2. Reflections focused on the impact of: 1) embracing a learning-based project culture; 2) adverse childhood experiences and trauma-informed practices (focusing on the four trust-related principles reflected in prior interviews: trustworthiness, safety, transparency, peer support); 3) strength-based approaches (resiliency focused) and the Community Resiliency Model; and 4) direct community involvement in ‘*the work.*’ We specifically queried community involvement in the initiative, as this took an increasingly formalized approach over the course of Innovation 2 through hiring staff with lived experience from the community to serve as a bridge between the community, community agencies and DMH, particularly in historically underserved cultural and linguistic communities. Staff included Peer Navigators (transition age youth who were the same age as youth populations served by some of the initiative’s strategies), *Promatores de Salud* (health navigators traditionally from Spanish-speaking communities), and Community Ambassadors (lay mental health workers who live in the communities they support), forming a network to educate the community about public health issues (including, but not limited to, COVID-19) and connect community members to services. Agency leads were also asked in what ways their project experiences changed their concept of mental health services and/or community health and well-being. Example questions included: *How do you define trust? What do you think are the key elements of trust-building within your (a) agency/organization, (b) partnership and (c) community? How has the concept of resiliency or the Community Resiliency Model changed your thinking about the community and how you connect with your community? What would you recommend to others as the key elements of capacity building and successful community engagement?*

### Data analysis

We employed a Grounded Theory methodology, incorporating iterative, multi-layered coding processes for all three interview waves [48–50]. As part of our ‘*First Cycle’* coding methods, two to four Team members (minimally including one PhD, and one BS, level coder) created relevant codes for the question topics and categories, guided by theoretical considerations and emergent themes in the interview content. For each annual interview set, qualitative data from 36–40% of the interviews were initially open coded by multiple team members using First Cycle Coding Elemental Methods (*Initial*, In Vivo, and *Descriptive Coding*), along with *Focused Coding* to develop and illustrate salient categories (given the semi-structured nature of the interviews) and follow-up *Memo Coding* in the form of shared written comments, impressions and ideas among team members [51]. While *Axial Coding* methods are considered ‘*Second Cycle*’, our interviews were both constructed and analyzed with attention to factors that might explain variation along dimensions, or across different levels (e.g., how strengths or obstacles are expressed within an agency, across a partnership, within a community, or between partnerships and the broader community). In each year, the codebook was expanded upon, adding new codes as needed, providing new exemplary In Vivo supporting codes, and crafting decision rules to facilitate consistency and reliability within and between coders, as well as create an audit trail.

After all coding questions were addressed and discrepancies were reviewed by multiple staff members, each year’s final codebook was uploaded into MAXQDA Plus software (VERBI Software, 2020), which was then used to code all transcribed interviews including interviews open-coded by multiple team members that were then reconciled across coders. Additional *Memo Coding* occurred within MAXQDA, and notations were shared and discussed among team members. Two team members (PhD, BS) were involved in the qualitative coding and oversight for interviews from all 3 years, with the BS-level staff member being the principal final coder across all years. Secondary coding reviews of randomly selected interviews (from each coder when there were multiple coders) were conducted by a PhD level staff member after MAXQDA coding was completed. While MAXQDA reliability was 88.7% among coders, as each emergent theme was further worked up using *First* and/or *Second Cycle* coding methods, Team members debriefed together and worked towards a consensus in a collaborative way.

We employed *Process Coding* to operationalize and classify key themes and findings related to the behavioral facets of trust-building in an action-oriented manner. We employed inductive (data-based) *Values Coding* to capture values, beliefs and attitudes that reflected participants’ worldviews from their own perspective (i.e., from the “emic” participant perspective, rather than capturing the coders’ perspective) [52]. Exemplary quote selection was part of an iterative process of independent selection, comparison, discussion and consensus-building among team members. Coding and emergent themes in each year aided in the development of questions for the interview guide in subsequent years.

### Post-hoc reflection on data depiction

After coding and interpretation was completed, we looked for useful ways to depict our findings that would better support reflection and actionable learnings about trust for a broader audience outside of the scientific community, including community members and staff with lived experience. During this post-analysis and interpretation process, we identified Poorkavoos, Hatcher & Smith’s [54] labor-market based Wheel of Trust*,* which presented trust-building elements that were found in their research to increase trust in organizations, reduce staff turnover and improve employee engagement in a accessible format. While we embraced their visual format, our question development, interview guide, codebook construction, analysis and interpretation were not a priori influenced by their work.

## Results

**Fig. 1:**
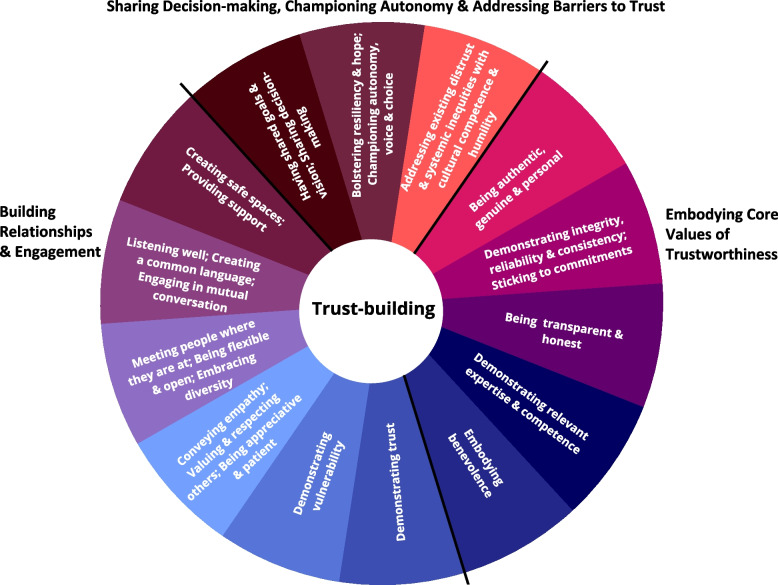
The community circle of trust-building Note: As derived from this community-based initiative, *Creating safe spaces* encompasses physical, as well as emotional and health, safety. *Providing support* includes increasing equitable access to resources, training and peer support opportunities

Throughout three waves of interviews spanning the initiative we identified three overarching trust-related themes, comprised of fourteen trust-building behaviors or practices, that we used to create the Community Circle of Trust-Building (see Fig. 1, Additional file 1 provides a Spanish-language version of this figure). The first theme was related to “Building Relationships and Engagement” and captures six implicit trust-building practices focused on building relationships. The second theme was related to “Embodying Core Values of Trustworthiness” and captures five explicit trust-building practices achieved through embodying values related to trustworthiness. The third theme was related to **“**Sharing Decision-making, Championing Autonomy, and Addressing Barriers to Trust” and captures three higher order, explicit collaborative trust-building practices, which reflect having shared goals, bolstering resiliency, and addressing systemic inequities. All three trust-building themes emerged organically during the baseline interview and were further explored during the midpoint and final interviews. Results are summarized below for each of the three waves of interviews and illustrate how trust-building practices represented by these three overarching themes often emerged together (e.g., relationship-building and being trustworthy are needed to effectively share decision-making). Additional supporting quotes are provided in Tables 2 and 3.

**Table 2:** Building relationships, engagement and embodying core values of trustworthiness

Relationship-Building	Embodying Core Values	Exemplary Trust-building Quotes
-Creating safe spaces and Providing Support -Listening well	-Being transparent	“I think that safety component also folds into **transparency**, because people when they’re when they feel like they’re in the loop of things and they know what’s going on, they tend to feel more safe. I think — that **transparency** has lent itself to keeping staff feeling informed and safe within their roles and within their teams… having reflective practice groups to provide safe spaces for people to chat; also getting supervisors trained on reflective supervision—trying to create those natural safe spaces for people to talk through any difficulties they’re having; and also just providing multiple opportunities, especially over the past year, for anonymous feedback through staff surveys asking people specifically about safety, physical safety, and emotional safety, and how they evaluate [our agency] on that.”
		“I think t**ransparency’s** a big one, and…we’ve been called out when that hasn’t happened…we were told: ‘You’re not being transparent. **We need more information. We want to know what decisions are being made, and why they’re being made**. And there’s a lack of transparency and that doesn’t make me feel safe and that doesn’t make me feel like I can trust you or others in this initiative.”
-Providing support	-Embodying benevolence	“…our community members [and] partners knowing that we will be there to support them, it’s a way of them trusting us. Even when the process of finding funding or resources, or decisions taking longer than expected, they continue to be our partners, because they **trust that we have their best interests on hand**."
-Creating safe spaces -Listening well; Creating a common language; Engaging in mutual conversation -Meeting people where they are at; Embracing diversity		“…because of the diverse community, I think there’s that culture piece, too, that [needs] understanding…because that’s also part of building trust is [being] able to create that space where people are able to communicate in their own language, in their own words…creating those welcoming spaces.… [And] cultures practice different ways of just healing and thinking about trauma, so I think it’s also understanding.”
-Providing support -Meeting people where they are at; Being flexible & open; Embracing diversity	-Embodying benevolence -Being transparent & honest -Demonstrating relevant expertise -Demonstrating integrity, reliability & consistency; Sticking to commitments -Being authentic, genuine & personal	“**being kind**, **transparent, having the best interest of our community members**, and being there and building those relationships at the different levels…with community members, with our staff, with our partners, with our funder.”
		“[W]hen I think of it on a one-on-one basis or that micro-sense of when they’re working with a participant, I think of it [as] this is someone who understands where this person is coming from in a way that others might not. They have this **depth to their knowledge**…that creates a really good foundation of trust with the people that they’re working with. And then, when I think of trust with the community or trust in a larger sense, I think it’s just **consistency**. It’s that **we’re consistently providing these services, consistently following through with what we’re saying we’re going to be helping you with, consistently being a face in the community through outreach so you know that we’re there and nothing has changed**.”
		“I think a lot of that comes from **authenticity**, and that’s why when I look at what we’re talking about, a lot of that comes from having good cultural understanding. A lot of that comes from being in a place where you’re able to support people, because you have that common knowledge.”
-Conveying empathy -Providing support	-Demonstrating integrity, reliability & consistency	“[T]rust is something that is…developed between two individuals, or two entities. [Y]ou do that by showing up, by those three Cs…the compassion, **the commitment, the consistency.** You’re a role model.”
-Demonstrating vulnerability -Meeting people where they are at; Being flexible & open		“I know that I have built trust, when someone is willing to be vulnerable with me, and vice versa. When I’m willing to be vulnerable with someone else, and open up to them, that’s when I know that there is a foundation of trust that has begun to build. I think that first invitation or share of vulnerability is that test of the ice when you go out on a frozen lake, and you tap it with your foot to see if it cracks.”
-Demonstrating vulnerability -Conveying empathy	-Being authentic, genuine & personal	“I think there’s vulnerability in creating a relationship of trust and **genuineness**…being able to be empathetic and I think [trust] is at the core of everything that we strive to do because without that trust, without that understanding…there’s definitely a barrier, a wall that’s created.…”
-Engaging in mutual conversation	-Being transparent & honest -Demonstrating integrity, reliability & consistency; Sticking to commitments	“ I think, definitely, **being open and transparent** as much as possible. Obviously, some things you can’t share immediately. But that’s been something that we’ve always done internally with our staff, just being as **transparent** as possible when we get updates from DMH—sharing those with our partners as well, so they’re not left in the dark. And I think for community it’s similar…really being open and sharing…if there’s changes in funding, changes in contracting, just making sure that we’re **consistently communicating with everyone with those changes**. Even when we have had to shift back and forth between the ways we’re providing services to the community, our teams are very good at communicating that and describing what that’s going to look like, so the families don’t feel kind of left behind. So, they’re constantly being reassured of—and communicated with about what’s happening. Not so much on a project level, but on a service level that they understand and interact with.”
	-Demonstrating integrity, reliability & consistency; Sticking to commitments -Being transparent & honest	“…the word confidence comes to mind. When I trust somebody I’m confident in them, when I trust somebody I feel that **I can rely on them, that they will bring me truth and honesty** whatever the answer might be, even if the answer’s not what I want, but I can trust them to be honest with me, I can trust them to be forthright, and so to me it’s that you can **count on them.**”
	-Demonstrating integrity, reliability & consistency; Sticking to commitments	“I think with trust there’s also **accountabilit**y…if we want to have that trust-building and engagement, there also have to be hard conversations about how others are being held accountable, and maybe how are we being held accountable to **delivering what we’re promising**…. Because if you promise something or you laid out something [about] what’s going to happen, and it doesn’t, then…that trust gets broken.”

For each quote, the most relevant part of the descriptors are provided for *Building Relationships* and *Embodying Core Values*. Underlined text reflects *Building Relationships and Engagement* practices, while bold text exemplifies *Embodying Core Values*

**Table 3:** Collaborative practices: sharing decision-making; championing autonomy and addressing barriers to trust

Sharing Decision-Making; Championing Autonomy & Addressing Barriers	Exemplary Trust-building Quotes	Building Relationships and Engagement	Embodying Core Values
Bolstering resiliency & hope; Championing autonomy, voice & choice	“I think there’s this whole concept of universal precautions and do we just assume that everybody has some traumas in their lives and **treat people with kindness** and let them know that when people have traumas, *we have resources, and you’re going to be okay. [Y]ou’re not broken, and we can help you. And then, just be ****kind and caring**** and ****look at people and recognize**** them instead of just handing them [off].”*	-Meeting people where they are at -Providing support	-Embodying benevolence -Being authentic, genuine & personal
	*“[B]eing there for community members when they need a resource already starts building trust, and then they’ll come back to you whenever they need some other resource.* [I]t’s an evolving relationship and it starts with that initial support or trust that that they felt… I also trust my staff and I know that our partners are doing what they’re supposed to and **my staff get to also be transparent** and provide that safety and **be kind to community members**. [T]hat’s how we end up being trustworthy to the community and transparent.”	-Creating safe spaces; Providing support -Being appreciative -Demonstrating trust	-Being transparent & honest -Embodying benevolence
	“I think it has to start individually first because then that kind of translates into the work that you do in the community. I was watching the staff interact with the community, have conversations and I would hear some of the messages that we had been talking about. [I]t’s the role modeling, it’s the being patient, **it’s the being transparent, the consistency part. [D]oing what you say you’re going to do is very important in the communities**…. **Don’t just tell me you’re going to do this; show me you’re going to do it. [T]hat goes back to leading by example.** [O]ur peer navigators, our staff, even our psychiatrists – all of them follow that same [recovery model]. It’s about holding folks accountable, *it’s that self-responsibility, allowing natural consequences.”*	-Providing support -Listening well; Creating a common language; Engaging in mutual conversation -Being patient	-Being transparent and honest -Demonstrating integrity, reliability & consistency; Sticking to commitments
-Having shared goals & vision; Sharing decision-making -Bolstering resiliency & hope; Championing autonomy, voice & choice	“When our CAN interns came onboard, they [said]’I don’t understand what’s being discussed at this meeting’, but I [said] ‘*I want your voice to be included. You have an input. Anything that’s mentioned, you can give your feedback, give your suggestions. [Y]our voice matters.’ [D]efinitely including them in the decisions that we’re making.* And, hearing their feedback since they are the feet on the ground working with community members.”	-Listening well; Creating a common language; Engaging in mutual conversation -Demonstrating Trust	
	“I may not be from a neighborhood that they’re from or may have grown up in a different way [than] they grew up, or have lived a different life than they have, I can still say: ‘*Hey, I got you. You know, we’re here for you, and you can continue to share with me.* Thank you for opening up to me, and I appreciate that. *[F]eel free to express that with me when you need to.* What are some things that we can do to talk about this more and make you more comfortable?’[T]o me, trust is vulnerability.”	-Creating safe spaces; Providing support -Listening well -Meeting people where they are at -Being appreciative -Demonstrating vulnerability	
-Addressing existing distrust & systemic inequities with cultural competence and humility	“I’ll use a specific example of why we think our role is so important to trust. *[T]he black community in [our area] are very untrusting of the government. [W]e’ve been looked at as the liaison, the relationship people, to try to defuse some of the distrust with the government, especially people that have been in the system for a really long time.* **So, our trust is through follow-through. Our trust is doing what we say…not disappearing. [B]eing there, even if we haven’t heard from them in a while. [W]e don’t promise our clients a whole lot and then not deliver.** *[T]hat has certainly been a challenge because people…have learned to distrust ‘the system’ for all of these years*.… It’s more [about] building a relationship and a bond, which takes time.”	-Meeting people where they are at; Being flexible and open; Embracing diversity -Being patient	-Demonstrating integrity, reliability & consistency; Sticking to commitments
	*“[T]rust, it’s a very powerful word and I think that is one of the reasons why even our young people are like ‘I don’t trust you. Why would I trust you? This entire system has done me wrong. So, why would I assume different from you?’* So, really being able to build that trust and see a young person say like **‘Okay, cool, you’re not trying to harm me,’ even when something goes wrong.** Really taking that opportunity to unpack that and dive into it.”	-Creating safe spaces -Meeting people where they are at	-Embodying benevolence
	“I think without that transparency…**folks don't feel safe...**it's also *being mindful of the power differences and dynamics in my role.*”	-Creating safe spaces	-Being transparent
-Championing autonomy, voice & choice -Addressing existing distrust & systemic inequities with cultural competence and humility	“We started a recent collaboration with a new organization, and they were doing what they were calling a ‘coronation’, which was basically a debutante program for young black girls… *[S]ome of my staff [were] like, ‘what are we doing?’ I'm like, ‘This might not be comfortable for us. We might not have called it a coronation and done a crown, but culturally, that works for them. Let's go with it.’[I]t turned out to be a fabulous collaboration* and that takes some convincing sometimes for my staff who's more traditional in how social services does its work. *I try to push them that it's okay to do it a little differently because, in that culture, that's how they do it.* **[Y]ou are trying to be a reliable member of the community.**”	-Meeting people where they are at; Being flexible and open; Embracing diversity -Creating a common language	-Demonstrating integrity, reliability & consistency

For each quote, underlined text reflects *Building Relationships and Engagement* practices, bold text exemplifies *Embodying Core Values*, and italicized text exemplifies *Sharing Decision-Making; Championing Autonomy & Addressing Barriers*

### Baseline Interview (Wave 1, following the initiative’s first year)

During the baseline interviews, trust-related themes conceptually emerged in two ways: (1) implicitly through building relationships and (2) explicitly addressing trust. Notably, these findings were not the result of queries specifically about trust or relationships, but rather emerged organically in response to questions about: a) unique aspects of their community; b) how partnerships assess community needs; c) their partnership’s main community goals; d) their primary roles and responsibilities as a lead agency; e) what authentic partnerships with the community look like; and f) any implementation lessons they had learned.

First, all but one agency lead described implicit ways that the seeds of trust were planted by meeting community members “*where they are at*” in terms of partnership members integrating directly into the community and engaging with community members in more naturalistic activities not linked to mental health services (e.g., knitting circles, cultural celebrations). The implicit trust-building behavioral practice of *meeting people where they were at* reflects “Building Relationships and Engagement**”** that authentically establishes rapport, fosters engagement, and increases opportunities to learn about community members’ needs over time, in a more natural fashion:

*“Partnership with the community is [about] making ourselves accessible to all members of the community. [N]ot just once they come in our doors, but really being out there at the parks, at the churches, at the flea markets, and not just being out there, but engaging with people. We don’t sit behind the table and the tablecloth that says “services” and just check our phone, right? We are in front of the table passing out the flyers, getting people to know who we are and engaging in different small talks, and saying ‘hi’ to their kids or [noticing] they have a puppy, all those things that are engaging. [B]ecause, if we do that consistently enough, we become part of that [community].”*

Meeting community members *where they are at* was linked to *creating safe spaces and providing support,* which in turn were conducive to community members willingness to express their needs:

*“[A] top-down approach is not going to work for really getting the voices of folks, but it's like: ‘are you open to sitting down next to this person and hearing them out and hearing what they're going through? [A]re you creating that space that's going to be comfortable for them to share that with you?’ Because, once you do that, then you can effectively be their partner….”*

Second, direct trust-building was explicitly referenced by the majority of agency leads as both demonstrating relationship-building skills and embodying core values and traits related to being trustworthy. These reflect central practices related to “Embodying Core Values of Trustworthiness.” Explicit trust-related themes arose in response to questions a-c above as well as questions about the biggest challenges faced in building community capacities, and what elements were necessary for community members to be empowered to ask directly for what they need. Explicit trust-building was described in terms of an ongoing process that fosters trust by being trustworthy (e.g., *demonstrating relevant expertise and competence, demonstrating integrity), championing autonomy* (looking at community members as whole people, not just someone with a problem that must be fixed), *voice and choice* and recognizing and *addressing existing distrust*.

*“[W]e'll have to build trust and rapport…but it's also [about] being able to adequately address the needs that will come up with community members.”*

*“[T]he goal would be to really truly have wellness within our community, but that also means learning to be trustful with the organizations that are here, because a lot of the community is not trustful. You have to [have] their trust before you can actually start implementing a lot of different things. So that would be our goal, to really increase [our] trustworthiness….*

*“[Y]ou have to look at the whole person.… [W]e understand that the relationship piece is the most important piece, like building that trust with the young people so that they feel comfortable to keep coming back and then to engage further in services.”*

While building relationships and being trustworthy were viewed as critical first steps before providing services or directing community members to resources, a third, higher-order, theme also emerged related to collaboratively working with community members by “Sharing Decision-making, Championing Autonomy, and Addressing Barriers to Trust.” The central practices of *addressing systemic distrust* while *bolstering autonomy* were explicitly reported as key elements of earning trust in the community.

*“It starts with us because they don’t trust big systems. [B]uilding that rapport and that relationship with us starts with being able to ask us what they want and us responding to, and praising, that action that they're taking. And then helping them take it to the next level. But it starts with us. We need to be able to create that space where they feel comfortable, because if they didn’t feel comfortable with us, or telling us what they need, how are we going to expect them to go to a town hall meeting and say I need this?”*

Building relationships, being trustworthy, supporting autonomy and repairing fractured relationships (e.g., addressing existing distrust) were the key elements of how partnerships approached implementing the initiative’s community capacity building strategies. These practices also aligned with the initiative’s use of a trauma informed lens (i.e., principles of safety and trustworthiness) [53].

### Midpoint Interview (Wave 2, end of second year)

During our midpoint interview, we asked how safety and trust continued to factor into the partnerships’ work with their communities and queried other key trauma informed principles. The three central themes of trust-building from our baseline interview were articulated. Agency leads reiterated the importance of “*meeting people where they are at*”, trust-building through embodying core values (e.g., being trustworthy) and addressing barriers to trust. Importantly, *being appreciative and patient* emerged as a key ingredient to community capacity building work and applied equally to partnership members. When agency leads were asked about how they intentionally addressed trust with their community, the need for their own patience emerged as an important skill that included an element of gratitude (i.e., appreciating that the little things often matter most):

*“…to develop those trusting relationships takes time, and there’s often a frustration that comes, because there’s this feeling of, ‘I’m not doing anything.’ Yet some of the most powerful things you can do is maybe break bread with someone or just be available or do something that’s totally unrelated.”*

*Demonstrating integrity, reliability and consistency* were necessary for fostering trust, and for creating safe and nonjudgmental spaces where community members were free to open-up about their needs:

*“I think we’ve done everything we can to keep our word. We’re not promising something that we can’t deliver on, because that really creates a rift. I’m trying to [create] a space where folks feel welcome. They feel seen. They feel heard. They feel like we’re not going to judge them.”*

One important way to put into practice the embodied value of *being authentic, genuine and personal* was by including community-facing staff who had lived experience similar to the experiences of the community being served (e.g., community members on staff who could be ambassadors of health knowledge):

*“I’ve seen how the Promotoras are able to connect with our community members, because they’ve either lived it themselves at some point…or have that level of empathy and compassion.”*

Incorporating staff with lived experience (consistent with the trauma-informed principle of peer support), and having staff embedded within the community, was noted to increase trustworthiness and reflect *integrity* as well as keep the importance of *addressing systemic distrust* and *sticking to commitments* at the forefront of partnerships’ awareness and practices:

*“[W]e're on an uphill battle in terms of getting people to trust systems, that's why [Community Ambassador Network] organizations, and even myself, as a community worker, I'm mindful of the systemic injustices that have taken place. So, I try to be particularly mindful about not over-promising, not being just yet another institution that lets folks down.”*

Several potential barriers to building relationships, being viewed as trustworthy, and collaboratively working within partnerships and with the community also emerged during the midpoint interviews. Specifically, communication-related challenges (e.g., lack of control over how and when information was disseminated even among partnership members) were recognized as potential barriers to safety and trust within partnerships that needed to be acknowledged and addressed:

*“I think barriers to safety and trust sometimes [are about] messaging in terms of probably how we communicate things, the timing [of when] we communicate. Sometimes it’s beyond our control. And change only happens at the speed of trust. [A]s we expand the partnership, the messaging around what…we’re trying to do and [how we are] supporting the community [is critical].”*

Finally, agency leads described the double bind of being tasked by the funding agency (DMH) to build relationships with, and engage, the community, yet they were not in control of the work, timing, or funding. The disconnections among funding sources, service delivery and community relations highlight the central importance of funding agencies also learning lessons from their own initiatives and community, including their partners. Specifically, there is a need for funding agencies to build healthy relationship (*demonstrating trust);* embody core values of trustworthiness (e.g., *demonstrate integrity, reliability, and consistency; stick to their commitments*) and share responsibility in more collaborative work that acknowledges historic distrust (*address existing distrust and systemic inequities)*. In other words, there is a need for funding agencies to recognize that community agencies and partnerships are not the sole source of trustworthiness – it must be embodied at every level of the service system, starting with the funding agency and its representatives:

*“…DMH is improving, to some extent, but historically [they have] left it to the community agencies to be the outreach trust-building entity. They’re behind the clinic seeing patients and providing psychiatric services. [But] there’s not a huge arm of trust-building, per se. It’s the community agencies that are out there doing the work and [building] the trust, right? So, when DMH says ‘go and do concrete supports, there's no limit, we don’t want one person going hungry, we don’t want one person losing their house, go, do it at all, you need more, why haven’t you spent this more, why haven’t you done all this stuff?’ And then they say, ‘oh well, actually, next month is the last month of [funding]. So, call back all your community partners and let them know [to] stop referring, because we're not doing that [anymore].’ It's us, the organizations, that have to say that to the community, not DMH.”*

### Final Interview (Wave 3, initiative wrap-up)

The three central trust-related themes that emerged in the first 2 years of the initiative were again echoed in interviews with agency leads as they embarked on their final year of the initiative. Agency leads recognized that an essential part of maintaining the relationships they had built over time required delivering on their promises (*demonstrating integrity, reliability and consistency*) so that communities understood that agencies had their best interests in mind (*embodying benevolence*):

*When I think of trust in the Innovation 2 project, I think of having a foundation of a relationship where you come to have some type of expectation that is satisfied overtime: [K]nowing when we’ll be in a specific place offering resources, know[ing] that those resources are valuable, and know[ing] that we have your best interests at heart.*

Responses continued to reflect a combination of intentional relationship-building skills (e.g., *engaging in mutual conversations*, *creating safe spaces, providing support, demonstrating vulnerability, conveying empathy*), core values (e.g., *transparency/honesty, integrity/reliability*) and more collaborative goals (*sharing decision-making, championing community members’ autonomy, voice and choice*).

“*[I]n general when we’re dealing with a crisis with a student or a member, we're very open and transparent, if we need to have them go to the hospital we have that open conversation of, ‘Here’s where we’re at. But we have choices. So how do we want to move forward, do you want to go this way or do we need to call in support?’ And again, that has to do with safety, being transparent, when you have an open conversation and kind of put the power back in the hands of the folks that you're serving, that safety kind of takes care of itself.”*

Trust was further characterized as dialogue that requires *providing support*, *engaging in mutual conversation, demonstrating vulnerability, being transparent and honest, bolstering resiliency and hope,* and *championing autonomy, voice and choice*:

*“Trust is about dialogue. It’s about being vulnerable, about sharing, about having an exchange. [T]hat's how I see operationalizing trust: people or community members having an honest conversation…, having the liberty to really express and communicate what is happening, what is really going on, and feeling like there's support in that.”*

It became increasingly evident across the interview waves that trust-related considerations were important for cohesion at every level: leadership, supervisors, staff, agency partners and the broader community. Supervisors’ role-modeling behaviors with their staff and trainees, and staff modeling (or embodying) skills in their interactions with the community emerged as important aspects of trust-building, specifically characterized by *Conveying empathy, Valuing and respecting others,* and *being appreciative and patient*:

*“…without there being judgment, shame or blame happening. That's something that I see with some of our teams where there is so much trust that people can just be themselves and say, ‘I don’t understand this. I need help.’ There’s that mutual support … It’s like, ‘I can freely express [that] I'm struggling right now, and it’s okay.’ [N]ow I see where [the Community Ambassador Network] is and... there’s so much trust in that team. [I]t has a lot to do with their supervisor leader because she makes herself available and is vulnerable about her needs and [the Community Ambassador Network] needs… [B]uilding that empathy for each other is also very much related to trust-building.”*

Reflective supervision is an approach comprised of several practices related to “Building Relationships” *(e.g., listening well; creating a common language; engaging in mutual conversation, creating safe spaces, conveying empathy)* and “Embodying Core Values of Trustworthiness” *(e.g., being transparent and honest).* Reflective supervision was reported as a particularly useful skill set:

*“[R]eflective supervision…[is] a great way to have that trust with your colleagues and your partners… checking in with them, making sure that they’re okay and…[that] it’s okay to say that ‘it’s not okay,’ We’re checking [in], we’re doing self-care.”*

*“One part of orientation that I always have when a new member comes on board is the commitments that I will make as a supervisor, and then the commitments that I will make as a community ambassador. [A]lot of that is around safety and empowering themselves, and making sure that we’re doing self-care and checking in with boundaries... I think that through the reflective supervision, we’re able to build on that trust because I am not just their supervisor or they’re not just a [Community Ambassador]…if they’re not feeling well or there’s a problem or a challenge with either one of our committees or a school that we’re working with, that we will have the support of our peers to continue empowering.”*

Finally, trust behaviors were seen as essential for navigating ongoing challenges due to the COVID-19 pandemic within the agency and partnership, including delivering on obligations to foster trust with colleagues, being transparent and working collaboratively with the community. Again, the little things were important: reaching out and making human connections.

*“It’s being able to like to rely on someone, being able to be candid with no judgement… I want to say that with my team, I have that trust and I think during this pandemic, it’s definitely elevated for us to be trustworthy because we’re working from home, trusting our peers that we’re completing a task, completing the projects that we’re working on and same with our partners, where we check in and we reach out to them and…also having that trust established with our community members and reaching out to them.”*

*“One of the things that we're really big on with [Transition Age Youth] is being transparent not just with each other, but also with the folks that we're serving, not trying to create this dynamic of ‘We're here and you're there,’ but really kind of normalizing and making a human relationship connection with the folks that we're serving.”*

### Putting it all together: The community circle of trust-building

Taken together, the three waves of interviews, spanning the initiative implementation until its final year, provided a contextual and theoretical roadmap for trust-building within partnerships, as well as between partnerships and community members in a large urban setting. Figure 1, the Community Circle of Trust-Building, provides a visual representation of our findings, and includes fourteen trust-building elements grouped into one of three conceptual categories reflecting the three overarching themes that emerged during the interviews. While behavioral practices (Building relationships and Engagement) and trustworthiness traits (Embodying Core Values of Trustworthiness) appear foundational for higher-order collaborative practices (Sharing Decision-Making, Championing Autonomy and Addressing Barriers to Trust), these three themes were highly interrelated, as evidenced by the co-occurrence of two or more themes in most quotes (see Tables 2 and 3). As noted in our Methods, the Wheel of Trust [54] provided inspiration for the visual depiction of our community-based trust-building elements.

There are other, broadly available, circles, or pillars, of trust and authenticity with various elements of trust and trust-building [55–58], but the Wheel of Trust’s eight trust-related constructs shared the most overlap with our findings. Specifically, all trust-related constructs identified by Poorkavoos, Hatcher & Smith’s [54] did emerge independently in our community capacity building project but additional trust-building elements, as well as overarching themes, arose within our data. We provide a comparison in Additional file 1, given that there was some conceptual overlap in our findings.

The first overarching theme “Building Relationships and Engagement,” includes six core trust-building behavioral practices. First, *creating safe spaces* at the emotional, physical and health levels, and* providing support,* such as skill training, resource access and peer support within the organization and with community members (e.g., community ambassadors) were seen as central elements for capacity building from a trauma informed perspective. Second, *listening well, creating a common language,* and *engaging in mutual conversations* were essential for effective communication internally (within agencies), across partnership members, with community members who were directly incorporated “into the work” (e.g., Transition Age Youth peer navigators) and with the broader community. Third, *meeting people where they are at, being flexible and open,* and *embracing diversity* were necessary ingredients for effectively engaging the community and staff. Fourth, *conveying empathy; valuing and respecting others;* and* being appreciative and patient* fostered mutuality and set the stage for collaboration. Fifth, *demonstrating vulnerability* furthered a sense of safety and openness to sharing. Sixth, *demonstrating trust* to foster trust in others emerged as central to mutuality amongst staff and community members, providing a foundation for identifying self-assessed needs and providing appropriate resources (community members) and training (staff).

The second overarching theme, “Embodying Core Values of Trustworthiness” captured five core values or traits that are foundational to a trusting relationship and reflect trustworthiness: 1) *being consistent*/*sticking to commitments*, and *demonstrating integrity, reliability and consistency*; 2) *being authentic, genuine,* and *personal*; 3) *being transparent* and *honest;* 4) *demonstrating relevant expertise and competence* and 5) *embodying benevolence*. Taken together, these facets of trust-building represent the core elements of trustworthiness that have also been identified in the broader, cross-disciplinary literature on trust: transparency and honesty; “ability” (competence, expertise); “character” which is typically reported to be composed of integrity and benevolence, and “consistency” (capturing reliability, dependability and commitment) [54, 59–63]. The third overarching theme of “Sharing Decision-Making, Championing Autonomy and Addressing Barriers to Trust” captured three collaborative practices. These collaborative practices are highly relevant to community- and equity-based work: 1) *having shared goals and vision and sharing decision-making*; 2) *bolstering resiliency and hope, championing autonomy, voice and choice*; and 3) *addressing existing distrust and systemic inequities with cultural competence and humility*. All themes and trust-building elements are captured by the exemplary quotes above and documented in Tables 2 and 3.

## Discussion

The present study captures an ongoing dialogue, in the form of three longitudinal waves of interviews with agency leads that spanned a community capacity building initiative in Los Angeles County from implementation to project conclusion. What emerged were common but central themes about the importance of 1) building relationships (e.g., engagement through meeting their community members “*where they are at*” physically within the community, emotionally, and in terms of their interests); 2) embodying core values of trustworthiness (e.g., authentically demonstrating integrity, reliability, transparency and benevolence), and 3) sharing goals and decision-making; championing autonomy, voice and choice; and addressing existing distrust and existing systemic inequities (i.e., barriers to trust). Each of the trust-building elements that fell under these three themes were described as an ongoing process that included relationships at all levels of stakeholders: the funding agency, the partnership, and the community. The practices that emerged as examples of these themes, were also important reflections at the individual level (e.g., the need for partnership members to be patient with themselves and other staff members when building and maintaining community relationships). As we followed this relationship-building thread over 3 years, valuable across-partnership lessons on the importance of trust-building at every stage of the initiative emerged: from inception, to navigating a pandemic and finally re-aligning with the initiative’s original strategies as the pandemic became more manageable with the availability of vaccines and reduced restrictions. For ease of reference, Fig. 1 provides a roadmap of important considerations for training, supervision, and effective engagement both within and between organizations and with the community members they serve.

Trauma-informed practices were central to the initiative and the principles of trustworthiness and transparency, safety, peer support and empowerment, voice and choice were all practices that emerged as essential to building trust with community members as a precursor to being able to effectively engage community members and connect them to relevant resources. However, there are no systematic guidelines for how to implement these ‘best practices’, particularly when working with large, diverse communities. These rich and thoughtful interviews from a large urban coalition provide some concrete steps for trust-building, related to relationship-building and engagement, consistently embodying core values related to trustworthiness, and creating broader collaborative goals among partnerships and community members. Consistent with Liu, Milojev, Gil de Zuniga and Zhang’s [2] description of trust as having universal and culture-specific elements relevant to interpersonal, organizational and institutional relationships and interdependencies, the trust-building steps that emerged from these interviews occurred across very different partnership and strategy configurations representing a range of communities and populations (e.g., densely urban downtown areas with significant Latinx and Black populations, rural and urban desert areas in Antelope Valley, large Cambodian population in Long Beach; strategies specific to transitional age youth 18–25 years old, LGBTIQA + youths, older adults ages 60 and older who are experiencing homelessness) and as such have potentially broader applications for funding agencies, clinical supervision, and community capacity building efforts that may be adaptable to different cultures, countries and environments. While the specifics of higher-order collaborative practices (sharing decision-making; bolstering resiliency; addressing existing systemic distrust/inequities with cultural competence and humility) may vary across different contexts, collaboration and power sharing are essential for productive, genuine partnerships with community members.

To extend these findings in tangible ways, there are several additional frameworks, models and/or trainings that support trust-building and may help guide others who are involved in equity-based community capacity building efforts. Trauma-informed practices are enhanced by specific neuroscience evidence-based actions that provide training on self-regulation and self-care skills that are beneficial for all stakeholders [64]. Self-care and self-compassion practices promote community health [65, 66], align with the Ottawa Charter for Health Promotion and community participation [67], and have been useful for community members in both developed and developing countries [68–70]. Self-care and self-compassion are also essential when working on the frontline of medical and mental health in communities where both acute traumas (e.g., assault, disasters) and longstanding adversity (e.g., community violence, poverty, discrimination) are prevalent, impacting both community members and those working for change and improved health [31, 71–73]. Frontline staff with lived experience are likely to experience both re-traumatization (i.e., a triggering of their own traumatic experiences) and secondary trauma as they witness the struggles of their fellow community members. Because mindfulness and similar self-care practices may be inadequate, perceived as patronizing or potentially discounting the severity of their adversity experiences, emphasizing social support, addressing systemic barriers to care access, and utilizing self-management practices borrowed from chronic illness care (e.g., self-efficacy enhancing skill development, lifestyle modifications, education) may be more openly received [74–76].

The reflective supervision framework directly mentioned by agency leads may provide important opportunities for self-efficacy enhancing skill development. This framework is trust-based, appropriate for gently validating staffs’ lived experiences that reflect the adversity exposure of their broader community, acknowledges that individuals are best able to problem solve in safe and supportive environments, models active listening skills, stresses the importance of empathy, and uses guided questions that build rapport, enhance communication, and facilitate each individual’s ability to arrive at their own conclusions and solutions [77]. While reflective supervision arose in mental health settings, the applications are wide, providing useful skills that work within organizations and are transferable to community interactions. This framework embraces shared power, with higher-order collaborative identification of needs and goal setting that align well with the *sharing decision-making* practice captured in the Community Circle of Trust-Building.

Similarly, Motivational Interviewing techniques, which were developed in the context of alcohol abuse treatment, are transferable to non-clinical settings and include empathic relational skills that support autonomy, self-efficacy and change motivation [78, 79]. Motivational Interviewing uses accessible mnemonics that aim to promote internal motivation/readiness for change, enhance listening skills, reduce ambivalence and/or commitment to the ‘status quo’, and guide insight-driven questions. These techniques may help elicit community needs when there is hesitancy or uncertainty on the part of community members, and increase the emergence of shared goals when there is a gap between public health concerns and community’s perceptions. ‘Rupture and repair’ practices from therapeutic alliance and clinical supervision models also provide a roadmap for addressing the inevitable mistakes (ruptures) that occur in all relationships and may even inform ways to address historic distrust and systemic inequities [80, 81].

The Community Resiliency Model is a strengths-based approach [82] that was explicitly incorporated to support autonomy and offset an exclusive “trauma-based” focus. This model arose from trauma-informed practices and was designed to address the impact (e.g., increased morbidity and mortality) of adverse experiences, by incorporating cultural and familial resources as well as filling in the gaps of trauma-informed models initially designed for traditional mental health service settings and trauma interventions designed to address single, past traumatic events rather than ongoing, widespread, co-occurring adversities (e.g., poverty, racial discrimination). The Community Resiliency Model also provides train-the-trainer options for community members. This type of community-accessible programming relates to the higher-order collaborative goals (‘Sharing decision making, Championing autonomy, and Addressing Barriers to Trust’) in the Circle of Community Trust-Building, particularly the *bolstering resiliency* and *championing autonomy* practices. While proprietary costs associated with training may be problematic, the ability of trained and certified community members to train other citizens helps to increase sustainability and offset the economic costs. It is also a useful framework for training agency and academic-community partnership staff, including those with lived experience, to provide direct support to community members and train a wide array of staff and citizens.

Similarly, the PEEPS relational framework was developed to enhance resiliency for youth by building positive peer and adult relationships, using a strengths-based approach, and addressing empathy and esteem [83]. This framework was drawn from the resiliency literature, designed to be accessible for a range of non-clinical community-based settings (e.g., adult mentor and parent-based programs, youth camps, school curriculum) and intended as a tool for wide application across cultures and countries. Leistner and Hart [83] also provide a list of resources for practical application to broadly support youth well-being.

Diversity related trainings (e.g., being an ally for the LGBTIQA + community, cultural competence, cultural humility), when embedded meaningfully and consistently at all levels of an organizational culture, are useful for *meeting people where they are at*; *being flexible and open and embracing diversity*, as well as *addressing existing distrust and systemic inequities with cultural competence and humility* [84, 85]. While the Community Resiliency Model and trauma informed practices tend to be broad, diversity trainings are the most culture-specific, requiring unique options for different countries that include relevant historical backgrounds to address deep rooted inequities. These trainings can be further strengthened within organizations by Inclusive Leadership strategies [86, 87] that value respect, cohesion/belonging, humility, cultural intelligence, collaboration and collective decision-making, while being aware of—and addressing—bias. Inclusive Leadership skills support *having shared goals and vision, sharing decision-making*, and *meeting people where they are at*, particularly with regards to *embracing diversity*, and may be an additional useful resource for organizations that work with directly with the community.

Finally, equity initiatives may be especially informative for supporting the collaborative practices that emerged in the present study. Resources include frameworks to promote shared decision-making, reflection practices that result in action, tools to address institutional distrust, and measures to assess outcomes in Community Based Participatory Research (Additional file 1 also provides resource links) [33, 34, 88–92]. Employing trust typologies (ranging from trust deficits/suspicion to critical reflective trust which captures trust mutuality and mirrors Rupture and Repair capacities) may increase awareness about the level of trust, and work needed to promote trust, in community partnership initiatives, while developmental stages provide more general reflection and collective practices that facilitate the transformation of communities along a pathway from engagement to ownership [88, 90]. Importantly, having a “place at the table” as advocated for in multi-stakeholder platforms and forums is a necessary, but not sufficient, condition to incorporating community members directly in initiatives that impact them [93]. True change requires addressing inequalities, supporting equity, ensuring participation, power sharing and even deferring to the community's decision-making [90, 93]. Mutual trust, requiring ongoing attention, effort and repair of ruptures, may be the foundation of these endeavors.

### Limitations and future directions

These supporting models and frameworks are meant to be a starting point in a broader conversation and will benefit from elaboration by authors representing different cultural viewpoints and countries. Similarly, although the proposed Community Circle of Trust-Building aligns with existing trust literatures from different disciplines [42, 59, 94–96], we are limited by data from agency leads engaged in a community-based initiative on the west coast of the US. These ‘trust-building’ data would be strengthened both by qualitative and mixed-method data from other community-based initiatives across the world, and learning about ‘trust’ directly from community members. The ‘learning culture’ practices used to share findings across partnerships and strategies in the initiative should be extended to community members. While individual partnerships in the present initiative engaged in sharing within their own communities, collaborative and collective reflection practices across communities and their members are likely to promote broader, more powerful actions [40, 92]. We acknowledge that trust-building can be misused and this increases the importance of authenticity, benevolence and transformational—rather than transactional—experiences alongside the co-creation of solutions and shared power [97–99]. We share these findings in the spirit of trust-building for positive outcomes, such as increased, equitable resource access, improved well-being, and a greater sense of belonging, which may have applications beyond community-based initiatives.

## Conclusions

Our journey through 3 years of interviews on a large-scale community capacity building project provided insight into trust-building as the foundation for collaborative relationships with community members. What emerged from these data were: 1) trust-building must be incorporated both within organizational cultures and across partnership interactions with community; 2) establishing trust takes time and patience – it cannot be rushed and must be continually earned; 3) trust-building requires mindful, intentional actions from funding agencies, leadership, supervisors and staff; 4) historical distrust must be addressed directly; and 5) building trust is an important, specific impact, independent of an organization’s mission, programming, or services.

Trust-building likely played a role in these partnerships’ ability to pivot from their specific, funded strategies, to providing for basic needs (e.g., housing, food) and establishing online technology and capabilities for families and students during COVID-19. As previously reported, these same partnerships made 14,000 service referral linkages for community members during the first 2 years of the initiative: 85% of community members followed through with their partnership-provided service referrals pre-Covid-19 compared to 87% follow-though during the pandemic [53]. These results offer hope that community members can be effectively reached by embedded organizations even during intense periods of health-related isolation, conflicting health information and local—or even large-scale—distrust.

Of note, one in eight individuals globally experienced mental illness in 2019 but there was a worldwide 26% increase in anxiety, and 28% increase in depression, during the first year of the pandemic alone, with women and young people most severely impacted [100]. Coupled with pre-pandemic data supporting the high prevalence of a range of childhood adverse experiences and trauma exposures across the lifespan [101–103] and evidence of increased financial distress, familial strain and violence exposure (e.g. domestic violence, child maltreatment) during the pandemic [104–106], the need for authentic relationships, a deeper understanding of our vulnerability and enhanced trust are all brought into high relief as essential for a healthy society. Warren et al. [107] provided a thought-provoking call to action just prior to the COVID-19 vaccine rollout: we should not expect or demand greater trust from disenfranchised or marginalized communities, but rather the onus is on individuals and organizations embedded in the community to earn trustworthy reputations. The COVID-19 pandemic has illustrated the need for increased trustworthiness, reliability and transparency among members representing governing bodies, the need for enhanced civility among citizenry, the need for collaboration between levels of governing bodies, and the central importance of local agencies in delivering services and addressing basic needs during emergency situations, as well as times of peace and prosperity.

## Supplementary Information

**Additional file 1.**

## Data Availability

UCSD IRB provided a waiver of informed consent to conduct the evaluation. However, this waiver does not include approval to release the qualitative data in its entirety. Access to the datasets generated and analyzed during the current study are restricted to protect the confidentiality of the participants. Reasonable requests for deidentified qualitative datasets can be directed to Todd Gilmer, PhD and are conditional on approval by the Los Angeles County Department of Mental Health.
