# Supplementary material for: Building trust: Leadership reflections on community empowerment and engagement in a large urban initiative
Source: BMC Public Health. 2023 Jun 28;23:1252. doi: 10.1186/s12889-023-15860-z (PMC10304359; doi:10.1186/s12889-023-15860-z)
Supplement: Supplementary file 1 — Additional file 1. [file 12889_2023_15860_MOESM1_ESM.docx]

Additional file 1

METHODS

Poorkavoos, Hatcher & Smith’s (2016) *Wheel of Trust* provided inspiration for an effective visual method of conveying the trust-building elements that emerged from our qualitative interviews.(1) The *Wheel of Trust* was developed by Roffey Park’s researchers, located in the United Kingdom and Singapore, whose charitable aims are described as supporting workplace health.(1) For the curious reader, we provide details to facilitate a comparison between our *Community Circle of Trust-Building* with their workforce based *Wheel of Trust*. However, all relevant details specific to our *Community Circle of Trust-Building* methods and analysis are provided in the main text.

RESULTS

The *Wheel of Trust* and *Community Circle of Trust-Building* were both derived independently from longitudinal qualitative interviews and share a visual format for conveying facets of trust-building. However, these independently derived reflections on trust-building differ in several important ways. First, the *Wheel of Trust* was developed specifically to address organizational trust, and not community capacity building efforts, community members’ resource access, community-level partnership development nor implementation of trauma informed practices with the organization or with its surrounding communities. Second, the *Wheel of Trust* was based on thematic analysis of workforce interviews with 17 individuals, over a period of seven months.(1) Our analytic methods (Grounded Theory), initiative (community capacity building through a trauma informed lens), design (longitudinal data collected over a period of three years, reflecting three naturalistic epochs: pre-pandemic, height of pandemic, and loosening of pandemic restrictions) and sample (agency leads from partnerships within a large urban county in the United States) are described in detail the main text. Third, analyzed data for the *Wheel of Trust* apparently did not reflect the thematic categories of trust-building that were independently derived from our multi-layered coding process (i.e., *Building Relationships and Engagement*; *Embodying Core Values of Trustworthiness*; and *Sharing Decision-making, Championing Autonomy and Addressing Barriers to Trust*). Fourth, regardless of thematic categories, Poorkavoos, Hatcher and Smith (2016) did not identify collaborative trust-building practices (e.g., *sharing decision making*, *bolstering resiliency and hope*, *addressing existing distrust and systemic inequities*) in their workforce data.

Poorkavoos, Hatcher & Smith’s (2016) *Wheel of Trust* reflected eight specific trust-related behaviors, that were not categorized or classified by superordinate themes: 1) *demonstrating trust*, 2) *being consistent*, 3) *sticking to commitments*, 4) *demonstrating vulnerability*, 5) *being transparent*, 6) *being personal*, 7) *appreciating others* and 8) *listening well*. These behaviors were found to increase trust in organizations, reduce staff turnover and improve employee engagement. While our independent findings overlap with all eight of the behaviors identified in the *Wheel of Trust*, our analysis revealed an additional seven trust-building elements relevant to community capacity building, as reflected in our exemplary quotes in the Results and Tables 2 and 3 in the main text. Further, some of the separate trust-building elements in the *Wheel of Trust* were reported together in our data (i.e., *being consistent* was reported alongside *sticking to commitments,* so we did not separate out these elements). Therefore, in our *Community Circle of Trust-Building*, the eight separate elements represented in the *Wheel of Trust* are instead captured in seven segments, which include further characterization that arose from our community-based data. Finally, as noted above, we also identified three core themes (akin to superordinate categories) that captured our 14 community-based trust-building elements, as reflected in the *Community Circle of Trust-building* (see Figure 1 in the main text; and Supplemental Figure 1 below, for a Spanish language version)

While Poorkavoos, Hatcher & Smith (2016) did not identify behavioral practices specific to collaborative trust-building efforts, there was overlap in some specific trust-building facets that we captured under our behavioral practices in *Building Relationships and Engagement* as well the values (traits) reflected under *Embodying Core Values of Trustworthiness*. For clarity, all elements identified in the *Wheel of Trust* also emerged from our independent qualitative coding of our own data and are reflected in the behavioral practices of relationship-building and values related to being trustworthy. As noted in their research summary, the behaviors traits identified by Roffey Park’s researchers were consistent with the broader literature relevant to psychological theory on why people trust others (<https://www.roffeypark.ac.uk/wp-content/uploads/2020/07/The-lived-experience-of-trust-report-with-covers.pdf>).

Our “*Building Relationships and Engagement*” theme includes six trust-building behaviors or practices, including the following four elements also independently identified in the *Wheel of Trust*: 1) *demonstrating trust*, 2) *demonstrating vulnerability*, 3) *appreciating others* and 4) *listening well*. Our qualitative coding indicated that ‘a*ppreciating others’* also specifically included *conveying empathy; valuing and respecting others;* and *being appreciative and patient*. Similarly, ‘*listening well’* emerged in our community-based data but was accompanied by *creating a common language* (particularly around trauma) and *engaging in mutual conversations*. Two sets of core behavioral practices, quite distinct from the *Wheel of Trust,* also emerged in our interviews with agency leads: 1) *Creating safe spaces* and *providing support*, alongside 2) *Meeting people where they are at; Being flexible and open*; and *Embracing diversity.* Therefore, the behavioral practices related to building relationships with, and engaging, the community were broader in scope than those identified in the organizational setting germane to the *Wheel of Trust*.

Our “*Embodying Core Values of Trustworthiness*” captures five values that reflect trustworthiness. Four traits identified in the *Wheel of Trust* overlapped with our community-based findings. In our data, however, two of the *Wheel of Trust* traits, *being consistent* and *sticking to commitments*, co-emerged together alongside *demonstrating integrity* and *reliability.* These behaviors were thematically similar and reported together in our qualitative interview data and are all reflected together as a single element. Second, the *Wheel of Trust* trait of *being personal* was also observed in our data but was accompanied by *being authentic* and *genuine*. Third, *being transparent* was identified in our data but was elaborated on as specifically *being honest* with the community and within agencies and partnerships. Fourth, our coding reflected the importance of *demonstrating relevant expertise and competence* and *embodying benevolence* but these facets of trust building were not reflected in the *Wheel of Trust*.

Spanish translation of the *Community Circle of Trust-Building*

In addition to our English language *Community Circle of Trust-Building*, we are providing a Spanish language version for use in community capacity building efforts.

**Supplemental Figure 1.** Community Circle of Trust-Building, Spanish Version


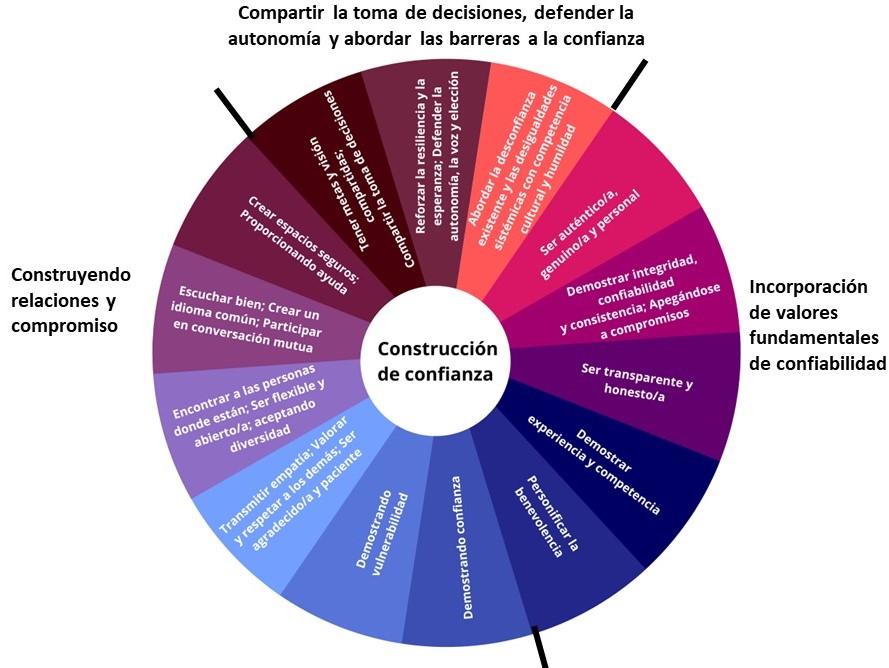


*Nota*: Crear espacios seguros incluyendo fisicos, emocionales y de salud medica. Proporcionando apoyo incluyendo entrenamiento de habilidades, acceso a recursos y apoyo entre compañeros.

ADDITIONAL RESOURCES

The Community Commons organization, which supports the US-based Equitable Long Term Recovery and Resilience plan(2) provides equity-based resources (<https://www.communitycommons.org/search?q=equity>) and a plethora of trust-related resources, including a toolkit for building institutional trust, resources specific to promoting patient trust, enhancing trust in science and vaccines, engaging people with lived experiences and research repositories: <https://www.communitycommons.org/search?q=trust>.

The National Academy of Medicine’s conceptual model for partnership development and community engagement around health equity considerations(3) is available at: <https://nam.edu/assessing-meaningful-community-engagement-a-conceptual-model-to-advance-health-equity-through-transformed-systems-for-health/>. Additional equity-based tools and resources specific to Community Based Participatory Research are available at: <https://engageforequity.org/tool_kit/>, including exercises related to sharing your core values to strengthen partnership trust (<https://engageforequity.org/tool_kit/claiming_your_principles/>). Collective reflection and evaluation resources to improve partnership practices are also available through Engage for Equity: resources for collective reflection and evaluation to improve partnering practices: <https://engageforequity.org/>.

REFERENCES

1. Poorkavoos M, Hatcher C, Smith A. 2016. The lived experience of trust: People’s stories of trust in the workplace. Horsham, West Sussex: Roffey Park Management Instituter.

2. Office of Disease Prevention and Health Promotion. 2022. Federal Plan for Equitable Long-Term Recovery and Resilience for Social, Behavioral and Community health. Rockville, MD.

3. Organizing Committee for Assessing Meaningful Community Engagement in Health & Health Care Programs & Policies. 2022. Assessing Meaningful Community Engagement: A Conceptual Model to Advance Health Equity through Transformed Systems for Health. *NAM Perspectives.* Commentary, National Academy of Medicine, Washington, DC. <https://doi.org/10.31478/202202c>.
